# Recent Advances in Smart Epidural Spinal Needles

**DOI:** 10.3390/s23136065

**Published:** 2023-06-30

**Authors:** Murad Althobaiti, Sajid Ali, Nasir G. Hariri, Kamran Hameed, Yara Alagl, Najwa Alzahrani, Sara Alzahrani, Ibraheem Al-Naib

**Affiliations:** 1 Biomedical Engineering Department, College of Engineering, Imam Abdulrahman Bin Faisal University, Dammam 34212, Saudi Arabia; 2 Department of Mechanical and Energy Engineering, College of Engineering, Imam Abdulrahman Bin Faisal University, Dammam 34212, Saudi Arabia

**Keywords:** epidural space, spinal needle, fiber Bragg grating sensor, epidural anesthesia, force measurements

## Abstract

Lumbar puncture is a minimally invasive procedure that utilizes a spinal needle to puncture the lumbar epidural space to take a sample from the cerebrospinal fluid or inject drugs for diagnostic and therapeutic purposes. Physicians rely on their expertise to localize epidural space. Due to its critical procedure, the failure rate can reach up to 28%. Hence, a high level of experience and caution is required to correctly insert the needle without puncturing the dura mater, which is a fibrous layer protecting the spinal cord. Failure of spinal anesthesia is, in some cases, related to faulty needle placement techniques since it is blindly inserted. Therefore, advanced techniques for localization of the epidural space are essential to avoid any possible side effects. As for epidural space localization, various ideas were carried out over recent years to provide accurate identification of the epidural space. Subsequently, several methodologies based on mechanical and optical schemes have been proposed. Several research groups worked from different aspects of the problem, namely, the clinical and engineering sides. Hence, the main goal of this paper is to review this research with the aim of remedying the gap between the clinical side of the problem and the engineering side by examining the main techniques in building sensors for such purposes. This manuscript provides an understanding of the clinical needs of spinal needles from an anatomical point of view. Most importantly, it discusses the mechanical and optical approaches in designing and building sensors to guide spinal needles. Finally, the standards that must be followed in building smart spinal needles for approval procedures are also presented, along with some insight into future directions.

## 1. Introduction

Various diagnostic and therapeutic procedures require physicians to perform a lumbar puncture (LP). It is a minimally invasive procedure that uses a spinal needle to puncture the lumbar subarachnoid space or epidural space in order to take a sample of cerebrospinal fluid (CSF) or inject drugs for diagnostic, therapeutic, or anesthetic purposes, respectively [1,2]. Surgical procedures, childbirth, and chronic back pain are some of the most common clinical applications. Other LP applications include measuring the fluid’s pressure, injecting medicine—such as painkillers, antibiotics, or chemotherapy, injecting a spinal anesthetic—to numb the lower part of the body prior to an operation, or removing some fluid to decrease pressure in the skull or spine [3,4]. These needles are also called epidural needles as they are injected into the epidural space. Therefore, localization of the epidural space is essential to avoid any possible side effects. Hence, high experience and caution are required to correctly insert the needle without puncturing the dura mater, which is a fibrous layer protecting the spinal cord. Currently, the loss of resistance technique is being used for LPs. However, this technique depends on the physician’s experience in correlating the force exerted on the tip of the needle to the specific tissue underlying the punctured area. Hence, failure of spinal anesthesia is, in some cases, related to faulty needle placement technique [5], given that the needle is blindly inserted. Due to the critical nature of the procedure, LPs display a failure rate of 28% [2,6]. Moreover, one-third of patients suffer from headaches following the LP that are sometimes accompanied by nausea, tinnitus, hearing weakness, fast heart rate, low blood pressure, and vomiting for a couple of days following the procedure. This is quite understandable as there is no real-time monitoring of the needle position [7,8]. As for epidural space localization, scientists have proposed various methodologies based on mechanical and optical schemes for the identification of the needle position, as will be explained in detail in this paper [2,9,10,11,12,13,14].

The spinal needle is needed in a variety of medical procedures, especially for anesthetic and diagnostic purposes in serious health conditions. Spinal anesthesia is also used as pain relief in surgeries such as labor surgeries. As mentioned, the spinal needle is also used to take samples of CSF to diagnose different diseases [2,15,16]. Hence, CSF can be utilized for the treatment of different diseases. For instance, Acute Lymphoblastic Leukemia (ALL) is one of the most common cancers in children. Hence, for diagnosing and treating this type of cancer, LP via catheterization and drug delivery plays a crucial role [2,15,16]. 

Multiple sclerosis (MS), encephalitis, Alzheimer’s disease, tuberculosis, subarachnoid hemorrhage (SAH), meningitis, fungal infection, bleeding in the brain, encephalitis, and more are just a few of the CSF diseases that can be accurately distinguished through an exhaustive examination [17]. Additionally, the pressure of the CSF is measured using a nanometer along with a spinal needle to investigate various diseases of the body. The measurement of CSF pressure can be used to predict the incidence of major intracerebral masses, the degree of cerebral edema, and the development of obstructive hydrocephalus. The high failure rates of lumbar and thoracic epidurals, as well as the incidence of inadequate anesthesia reported in cesarean section patients, indicate the need to develop improved techniques to minimize the negative impacts of such complications [18]. The pressure of CSF varies significantly, which can cause a range of symptoms such as headaches [18]. Furthermore, a spinal needle is also utilized to administer anesthetics prior to surgery as well as during labor and delivery to block and reduce pain [11].

The main goal of this paper is to remedy the gap between the clinical side of the problem and the instrumentation engineering side by examining the main techniques in building sensors for such purposes. The manuscript is organized as follows: First, the anatomy and physiology of the spinal cord are briefly explained along with the clinical needs of spinal needles in Section 2. In Section 3, we discuss the historical background of spinal needles. In Section 4 and Section 5, we present various mechanical and optical approaches to building sensors, respectively. In Section 6, we present a comparison of the different systems. The standards that must be followed in building spinal needles for approval procedures are discussed in Section 7. The paper is finally concluded, and some future directions are discussed in Section 8.

## 2. Anatomy and Physiology of Spinal Cord

Identifying the epidural space is critical to ensure patient safety; an understanding of the basic spinal cord anatomy is a must. The spinal cord is a crucial part of the Central Nervous System (CNS), and one of the most important parts of the vertebral canal, which elongates from the bottom of the brainstem (medulla oblongata) to the first lumbar vertebra (L1). In the spinal cord, nerve signals are carried throughout the body, and these signals perform three major functions: regulating body movements, sending signals to the brain, and managing reflexes [11]. It consists of five regions: cervical, thoracic, lumbar, sacral, and coccygeal. A different shape or thickness is present in each of these regions, as well as a specific number of pairs: 8, 12, 5, 5, and 1, respectively. 

**Figure 1:**
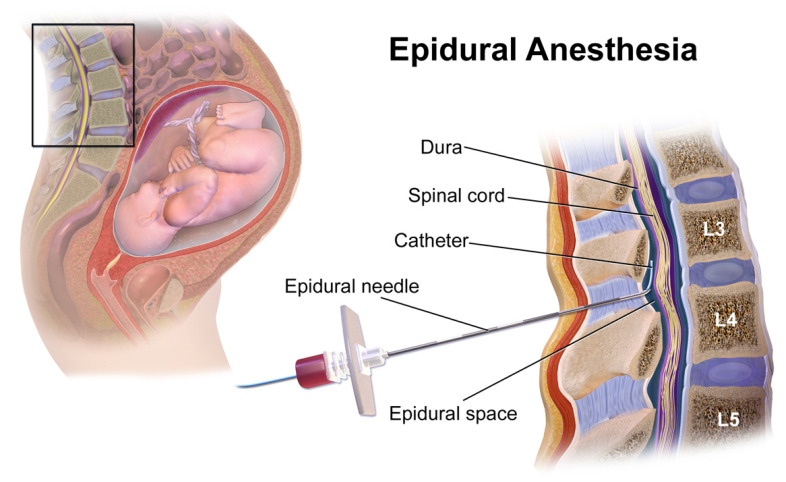
The anatomical cross-section of the spinal cord. Reprinted with permission from [20].

Figure 1 illustrates the anatomical cross-section of the spinal cord. The layers that cover the spinal cord are skin, subcutaneous fat, muscle, Interspinous Ligament (ISL), supraspinous ligament, ligamentum flavum (LF), epidural space, dura mater, arachnoid, and pia mater from outer to inner, respectively [19]. ISL is a thin and short structure that connects adjacent spinous processes and contains sensory nerves [1]. Moreover, it consists of a mix of collagen and elasticity; compared to the thoracic region, it is thicker and broader in the lumbar region. ISL meets the LF anteriorly and the supraspinous ligament posteriorly. According to Ref. [1], the direction of the ISLs fibers is parallel to the spinous processes with a slight curvature. 

Based on the spaces and measurements, the widest ISLs were found at L3/L4 and L4/L5, whereas the greatest lengths were located at L1. In terms of tensile strength, the ISL had a mean of 162.33 ± 38.36 N at L1/2, 85.67 ± 37.25 N at L2/3 and 79.00 ± 27.31 N at L3/4. With this measurement, physicians can predict the severity of injuries and hyperflexion caused by trauma. However, the main function of the ISL is to prevent hyperflexion and stabilizers of the spine [1,2]. 

The supraspinous ligament is composed of white fibrous tissue and connects the tips of the spine from the seventh cervical vertebra (C7) to the sacrum. Moreover, it has some resistance that affects the forward movement of the needle; on the other hand, the ISL is dense enough to keep a needle in place [1], while the LF consists of 60% to 80% elastic fibers, extending from the sacrum to the second cervical vertebrae (C2). Thus, it is considered to be the highest level of tissue elasticity in the human body. Due to its elastic fibers, the LF avoids buckling into the vertebral canal and the intervertebral foramen. Moreover, LF is present in each vertebral canal and connects the laminae of adjacent vertebrae. In addition, ligaments become thicker as they progress from cervical to thoracic to lumbar [21]. 

Dura mater is a dense collagenous connective tissue that encloses CSF and protects the brain and spinal cord. Epidural space is a layer of fat and blood vessels over the dura mater, which separates it from the vertebral canal. LF is a layer just above the epidural space. So, the epidural space is posterior to the dura mater and anterior to the LF [22]. Arachnoid mater consists of collagen and elastic fibers and is responsible for CSF metabolism, and subdural space separates dura mater from arachnoid mater. Moreover, subarachnoid space separates the arachnoid from the pia mater and is filled with CSF, which is used for microbiology and biochemical examination [22]. 

## 3. History of Lumbar Puncture and Spinal Needles

Lumbar puncture is one of the most frequently performed invasive clinical procedures today. The procedure is carried out for both diagnostic and therapeutic purposes. The history of LP dates back to late 1890 and is said to be discovered by Heinrich Quincke [23,24]. Prior to performing such a procedure on human patients, Quincke carried out research on rabbits, dogs, and human corpse, which helped him learn more about the anatomy and physiology of the spinal region [24]. Through this work, he understood that the ventricles in the brain were in continuous contact with both the cerebral and spinal CSF spaces. This key discovery led Quincke to begin experimenting with LPs in an effort to treat the symptoms of hydrocephalus in children, which is a neurological disorder caused by the buildup of CSF in the brain’s ventricles [25]. This anatomical and physiological uncovering was a scientific breakthrough since, prior to this discovery, Quincke drained CSF by drilling holes directly into the skull [24]. His new therapeutic procedure consisted of draining some of the excess CSF for pressure relief, which consequently alleviated the children of intense headaches associated with this disorder. He performed the procedure by inserting a thin, hollow needle into the subarachnoid space between L3 and L4. 

The first ever LP performed for this purpose was carried out in 1890 on a comatic 21-month-old child who was believed to have tuberculous meningitis [24]. The child exhibited a great improvement in symptoms after undergoing the procedure using the LP to relieve the buildup of CSF. Quincke later performed the same procedure on an adult with hydrocephalus and greatly reduced his headaches. Although Quincke began with therapeutic purposes, he soon realized the potential of LPs for diagnostic purposes and shifted his efforts to that area. After a few years of research, he published a paper describing his findings on the cell count and protein levels in CSF. He also identified bacterial presence in the CSF and discovered that patients with purulent meningitis exhibited decreased glucose levels in CSF, while those with tuberculous meningitis appeared to have tubercle bacilli in their CSF.

The first spinal syringe was invented by an Irish surgeon known as Francis Rynd in 1844, who created the first hollow needle [26]. Rynd’s design was improved 10 years later by the French physician Charles Pravaz, creating the first practical metal syringe. The needle tip was then enhanced by Daniel Ferguson, creating better penetration of the skin through the addition of an oblique sharp tip [27]. Ferguson’s design, however, did not allow for deep tissue penetration. Therefore, Alexander Wood improved Ferguson’s design in 1854 to overcome this obstacle [26]. 

Almost thirty years later, an American neurologist known as Leonard Corning redesigned Wood’s needle for better performance as he faced obstacles during his tests using Wood’s design [28]. Corning’s needle incorporated a shorter bevel, screw, and needle stop that allowed him to control the depth at which the needle reached to avoid accidentally puncturing deeper tissues and CSF leakage. A needle similar to Corning’s was used by Quincke during his studies, called the Quincke needle. Many scientists continued experimenting with the spinal needle design and concluded that the smaller and more round needle tips performed better than the blunt-tip design and resulted in less CSF leakage and trauma to the patient [29]. Today, many spinal needle designs exist but one of the most popular designs is the updated Quincke needle, which is composed of an inner and outer needle. The inner needle is placed to support the thin, hollow outer needle during the puncturing of tissues. Once the needle is in the desired position, the inner needle is removed to allow for the insertion of a catheter to administer medications or collect CSF samples.

## 4. Mechanical Approaches and Haptic Simulators

It has been demonstrated that using spinal needles with non-cutting tips improves safety and lowers the incidence of post-dural puncture headache (PDPH) [30,31]. A 19.5-gauge Special Sprotte TM epidural needle with a solid bullet-shaped tip and a lateral aperture for the passage of an epidural catheter was previously developed [31]. Studies comparing the 18-gauge Special Sprotte TM epidural needle to a 17-gauge Tuohy needle revealed decreased cerebrospinal fluid leakage and PDPH after accidental dural puncture (ADP) [32]. These observations led to the development of a novel 17-gauge pencil-point epidural needle with a rounded pencil-point tip devoid of cutting edges and a lateral aperture for the insertion of an indwelling catheter or spinal needle [31].

A significant challenge in such a procedure is hitting a spinal bone during insertion. In most cases, the practitioner must retract the needle and direct it in a different direction, prolonging the operation and aggravating the patient. The insertion of epidural needles is aided by a variety of technologies, including fiberoptic and ultrasound methods. The use of ultrasonography complicates a technique that is generally conducted without assistance and necessitates additional training [33]. Both real-time and pre-procedural ultrasound guiding of the procedure is possible using ultrasound imaging of anatomical landmarks. The depth of the epidural space (EDS) and the angle of the needle insertion can both be determined by ultrasonic sensors [34,35]. Real-time ultrasonography, as opposed to preprocedural scanning, would not be impacted by changes in the patient’s position and may therefore be more accurate [2]. Fiber optic techniques are only capable of being used at a distance of around 2 mm, which is too close for them to help prevent bone strikes [36,37]. In the next paragraph, we will discuss how this challenge can be addressed. 

**Figure 2:**
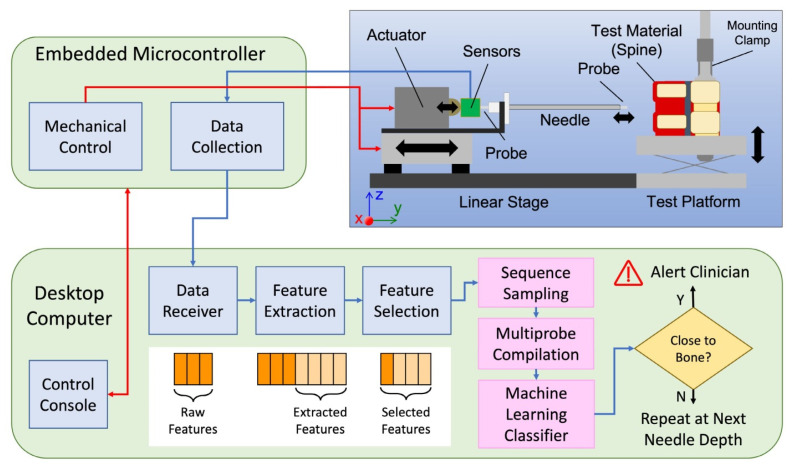
An overview of the developed data collection test bed for the epidural needle guidance system. Reprinted with permission from [39].

It has been established that viscoelastic measurements of soft tissues over bones vary depending on the thickness of the soft tissues [38]. This entails presenting tissue with a mechanical stimulus and analyzing the resulting force and motion responses, which differ significantly between various biological materials. As an application of this methodology, Simpson et al. presented a technique for detecting an imminent bone strike ahead of time [39]. This method allows the needle to be preemptively steered and the strike to be prevented by measuring the mechanical properties of the tissue prior to epidural needle insertion. An epidural needle insertion table-mounted device was developed, which was attached to the back of the needle during the insertion process as shown in Figure 2. This method could predict the needle’s proximity to the bone by 3 mm before it actually touches it. The authors used ex vivo tests on pig spine and observed that the system can reliably determine if the needle is at and within 3 mm of the bone. This distance does not provide the 5 mm of forewarning that the authors suggested would be necessary for use in clinical epidural needle placement.

There has been an increase in the use of haptic devices in medical simulations in the last few years, providing an accurate way of re-creating the feeling of surgery via simulation [40,41]. Since the initial release of simple epidural simulators in 1980, they have been enhanced and developed. The ability to graphically visualize the needle among the tissues is made possible by computer-based simulations. Examples of such simulators include Mediseus [42], which uses haptic feedback devices enclosed in portable cases and connected to laptops with graphic displays. The lack of measured data for force feedback has been highlighted as a weakness of Mediseus and other devices [42,43]. 

**Figure 3:**
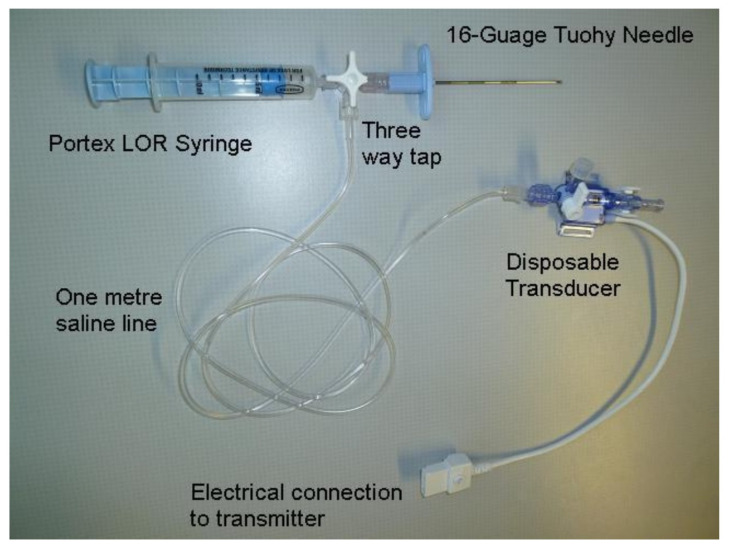
The disposable epidural pressure measurement system. Reprinted with permission from [13].

**Figure 4:**
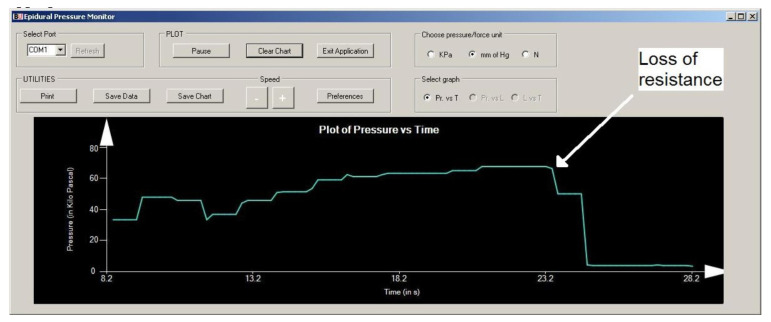
Screen print of the software interface to monitor and record pressure. Reprinted with permission from [13].

Figure 3 depicts an instrument that measures depth and pressure that has been presented for the first time [13]. Throughout epidural insertions, the data for pressure and depth were successfully recorded, as shown in Figure 4. A distinct graph was created for each needle insertion. The worst-case error in all trial runs was found to be within 3 mm, and the overall reliability was 97.8%. According to anesthetist feedback, porcine tissue does have characteristics similar to human tissue and the graphs obtained following the porcine insertion of the needle are similar to graphs obtained from previous human insertions [25]. The graphs that are produced in the majority of cases demonstrate when the low resistance (LOR) happened as the needle reached the epidural space. A peak on the graph may have occurred in some cases due to the needle hitting bone, but when re-angulation of the needle and subsequent advancement of the needle were carried out, the loss of resistance was still achieved [25].

A number of groups are currently working on developing simulations of the entry of needles into soft tissue. An insertion technique based on measured planar tissue deformations and needle insertion forces is investigated by DiMaio and Salcudean [44].

## 5. Optical Approaches

Among various optical sensors, Fiber Bragg Gratings (FBGs) are well known for their high sensitivity [45]. More specifically, they can be integrated into optical fibers and utilized for a wide spectrum of applications. They are fabricated by exposing the core of the fiber laterally to a certain pattern of powerful laser light. In turn, this leads to a production of a permanent increase in the refractive index of the fiber’s core. Therefore, there is a certain index modulation following the exposure pattern. The period of grating and the developed effective refractive index define the Bragg wavelength. This wavelength experiences a shift when external stimulations such as strain variations or temperature are applied to the fiber environment. Such a shift in the Bragg wavelength typically represents the sensing measure of the FBG [45]. Hence, designs of smart needles for epidurals have adopted the FBGs [7,8,14,46,47,48] and have been successfully utilized in some pioneering papers using a phantom and in vivo as well. 

In one of the interesting studies, the authors investigated a selection of needles with different gauges that are typically utilized by surgeons [8]. Based on the various tissue layers, they found that the required force for puncturing is rather different. Hence, the authors presented a comparative analysis using four unique gauges of the spinal needle. This evaluation has been carried out in real-time; hence, dynamic force monitoring has been achieved for the device that utilizes a Fiber Bragg Grating sensor. Next, the Fiber Bragg Grating Force Device (FBGFD) is employed to determine the dynamics of the force on various spinal needles with different gauges during the lumbar puncture procedure that is conducted using a simulator model and later on a human cadaver specimen. 

**Figure 5:**
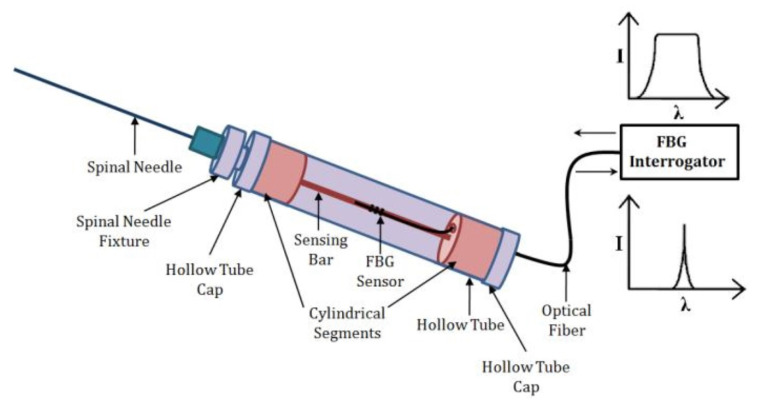
Schematic of Fiber Bragg Grating Force Device. Reprinted with permission from [8].

**Figure 6:**
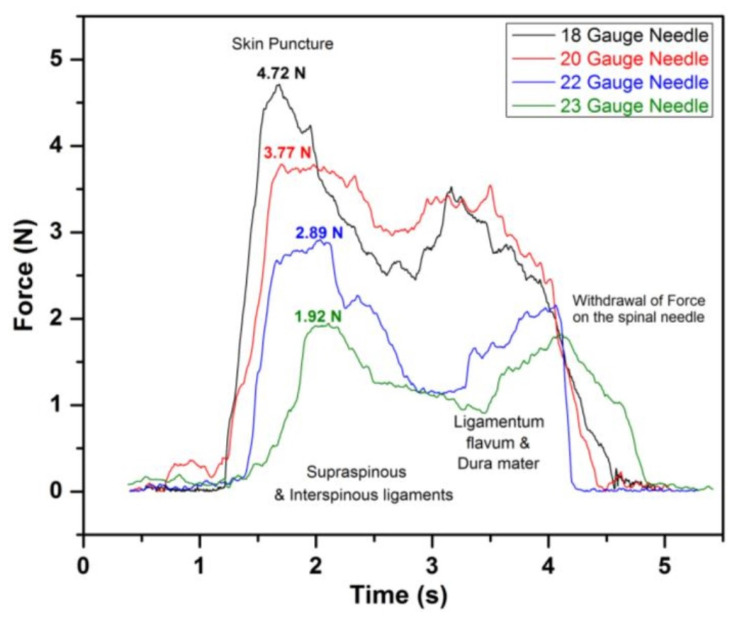
Force analysis for various regions traversal using different spinal needles on cadaver specimens. Reprinted with permission from [8].

The FBGFD consists of a sensing bar of 0.9 mm diameter and 40 mm length. As depicted in Figure 5, it is secured with two segments that have a cylindrical shape. Moreover, the FBG sensor was attached to the surface of the sensing bar. Figure 6 shows the force analysis of various regions using different needles on a human cadaver specimen. The four responses correspond to the traversal of four spinal needles puncturing different tissue regions. For the spinal needle of 18 GA, it is evident that the force on the spinal needle sharply increases and reaches a peak force of 4.72 N and 1.92 N for the 23 GA spinal needle. The resistance then decreases for further traversal of the needles, and it is less than the skin puncture resistance. Interestingly, the FBGFD offers dynamic data of force variation on the spinal needle with a resolution of 0.021 N for the FBG sensor. This study could serve as a guide for the suitable gauge of the spinal needle in order to minimize the post-puncture effects on patients.

**Figure 7:**
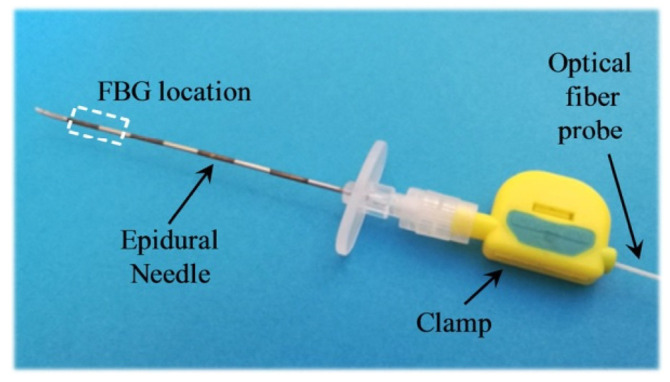
Picture of the integrated device in the Tuohy needle. Reprinted with permission from Ref. [48].

In another in vivo study, the authors demonstrated how optical fibers can be used to guide the placement of needles during epidural anesthesia [48]. In this study, two FBGs have been integrated into a Tuohy needle with Bragg wavelengths of 1557.7 nm and 1543.6 nm, as shown in Figure 7. During the experimentation, a female pig (age 7 months, weight 70 kg) was anesthetized with an intravenous injection. In total, six needle insertions were manually carried out into L5/L6, L6/L7, and L7/S1 interlaminar spaces. 

**Figure 8:**
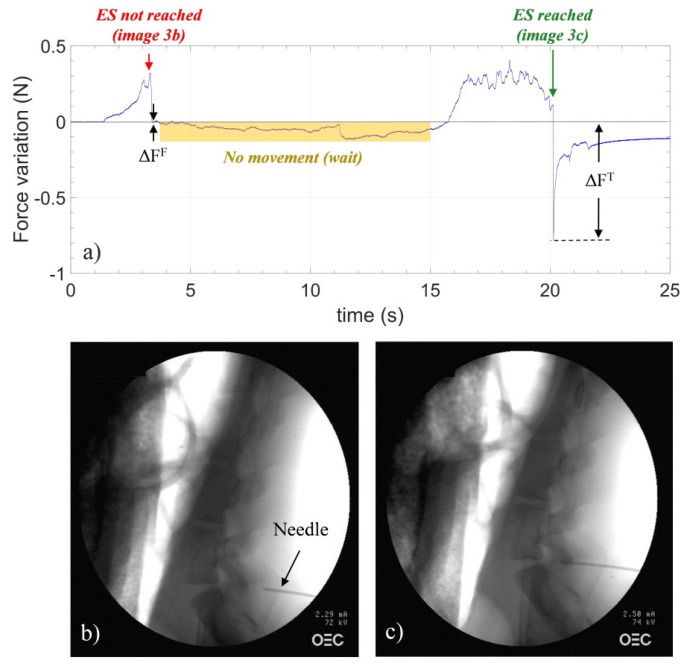
(**a**) Force variations versus time measured by HCP in L7/S1. The gray line represents the baseline (details in the text). X−ray images captured for monitoring the needle position during advancement: (**b**) wrong and (**c**) correct positioning of the needle. Reprinted with permission from Ref. [48].

Figure 8a shows the measured data using Hytrel coated probe (HCP) in L7/S1 [48]. Moreover, X-ray images were taken to capture the needle position during insertion for both the wrong position (b) and the correct position (c). As soon as the procedure started, an increase in the force was observed, followed by a force decrease that happened at about 3 s. Then, an X-ray image was taken while stopping the needle. The image shown in Figure 8b reveals that the needle tip is rather far from the epidural space. After ~15 s, the needle advancement was restarted until another sudden drop occurred at ~20 s. In this case, one can conclude that the needle tip actually goes through the LF. The needle enters the epidural space, inducing a fast relaxation of the probe and thus a force drop. The corresponding X-ray image was taken at that point as shown in Figure 8c and it confirms that the needle entered the epidural space. The authors of this paper found that the maximum distance between the trigger signal and the overall space identification uncertainty is 0.60 ± 0.35 mm. Considering that the epidural space depth is typically in the range of 4–7 mm in the mid-lumbar region, this device seems to correctly meet the need of placing the needle in position inside the space.

**Figure 9:**
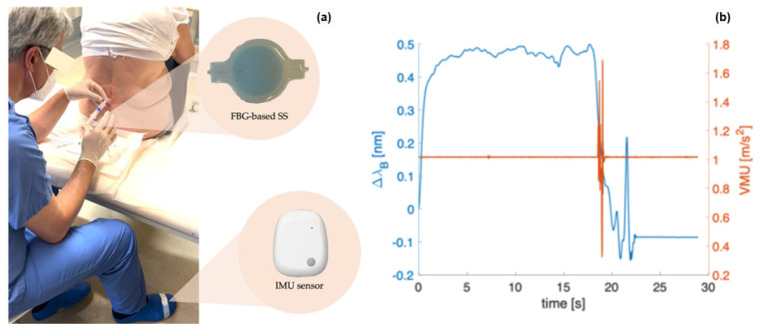
(**a**) Experimental set−up of one of the epidural procedures; (**b**) ∆λB (blue lines) and VMU (orange lines) signals as a function of time expressed in s for each of the patients enrolled. Reprinted with permission from Ref. [49].

More recently, another novel system containing an FBG implanted within a flexible silicone rubber was proposed [49]. This study assessed a device that was made of Dragon SkinTM 30, with a thickness of 8 mm and cylindrical in shape for loss of resistance (LOR) detection during epidural puncture. Unlike previously proposed solutions, this design principle supports clinicians without disrupting the standard procedure. Nevertheless, the mechanical properties of a particular rubber and its thickness used can impact the exact final properties of the system. Hence, the authors had to optimize the proposed soft system based on the right combination of materials and design. The feasibility of the soft system in clinical settings was evaluated by correlating its outcome to the clinician’s perception. In the first phase, as soon as the needle was inserted in the puncture site, the clinician would place the soft system on the syringe’s plunger, as shown in Figure 9a. To collect the measurements for the entire duration of the procedure, the soft system was attached to an optical interrogator at a sampling frequency of 1 kHz.

Figure 9b depicts the ∆λ_B_ and vector magnitude unit (VMU) signals versus time in blue and orange, respectively. The response collected from the soft system can be classified into three main periods: (i) a rapid increase in ∆λ_B_ as a result of the pertinent forces applied by the clinician in order to move the needle forward into different tissue layers; (ii) ∆λ_B_ values were above a certain threshold; (iii) an abrupt decrease in ∆λ_B_ when the tip of the needle crosses the ligamentum flavum and reaches the epidural space [49]. More importantly, the recorded data from the accelerometers measured in the VMU were estimated to be about 1 g during the procedure because the physician’s foot was fixed to the ground. Interestingly, once the LOR is felt, the authors observed a sudden change in the VMU due to the foot tap. As is evident from the plot, a noticeable peak in VMU takes place right after the sudden drop in the soft system output. Hence, this signal demonstrates the capability of the soft system to detect the LOR accompanying the needle’s position, reaching the epidural space. Moreover, four patients were enrolled in this study. The response of the soft sensor across the four trials showed the same behavior, characterized by a sudden drop in ∆λ_B_ when the needle tip crossed the ligamentum flavum and reached the epidural space due to the LOR.

**Figure 10:**
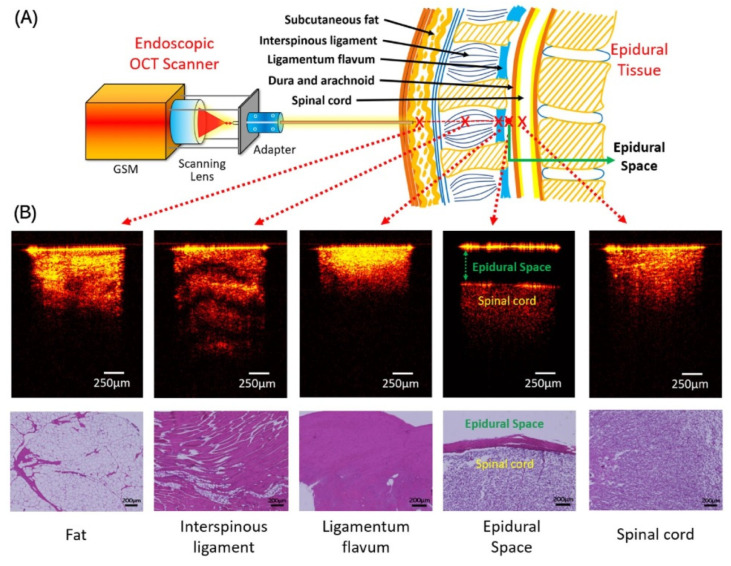
(**A**) Endoscopic OCT scanner setup and the representative OCT images of five epidural tissue layer categories. (**B**) Histology results of different tissue layers. Reprinted with permission from Ref. [12].

Another pioneering idea in use is endoscopic Optical Coherence Tomography (OCT) [12]. It was developed in order to image the different layers of the tissue when the needle tip advances. It was tested in porcine backbones. Moreover, in order to automatically process the imaging data for needle localization, a set of deep learning models was developed. The measurement system was built using the endoscopic OCT system and the schematic of the experiment is shown in Figure 10A. Figure 10B shows cross-sectional 2D OCT images of fat, interspinous ligament, ligamentum flavum, epidural space, and spinal cord. The epidural space was the easiest to identify due to the distance between the needle tip and the dura mater. The interspinous ligament stood out among the other four tissues for its clear transverse stripes and maximal penetration depth because of its thick fiber composition. Ligamentum flavum displayed increased imaging brightness near the surface and the shallowest image depth when compared to other tissue types. While the imaging depths of the spinal cord and fat were comparable, the imaging intensity of the fat was not as uniform as that of the spinal cord.

In Figure 10B, the matching histological findings were also presented. With the exception of fat, these tissues displayed various cellular distributions and architectures and corresponded well with their OCT results. In histology, pockets of adipocytes were seen in the fat tissue, but the OCT data did not clearly show this feature. This could be interpreted as the tissue compression that the authors used to mimic the clinical insertion scenario. In order to recognize the different layers of the backbone, a number of binary classification models were created. These layers include fat, interspinous ligament, ligamentum flavum, epidural space, and spinal cord. An average classification accuracy of 96.65% was provided by the classification models. While puncturing, it is crucial to keep a certain distance between the needle tip and the dura mater for safety reasons. Based on the OCT imaging data, regression models were created to calculate the distance. These models, which were built using the inception architecture, had a mean absolute percentage error of 3.05% ± 0.55%. Overall, these findings supported the technical viability of applying this unique imaging method to automatically identify various tissue structures and gauge the distances in front of the needle tip during the insertion of the epidural needle.

## 6. Comparison

Regardless of the considerable development of epidural spinal needles, there is still a tremendous need for high-accuracy, automated, and affordable techniques that can replace conventional manual-based epidural needles. The proposed methods face many challenges, including sensitivity, specificity, system stability, and calibration. Among the optical techniques discussed above, FBG and OCT were the potential candidates for achieving the goal of obtaining optimal automated epidural spinal needles. Table 1 summarizes different, recently developed techniques for epidural spinal needles. Nevertheless, a more specific and quantitative analysis should be evaluated for each of these techniques using quite a large number of subjects by giving the total number of punctures performed and the corresponding failure rate. 

## 7. Standards and Approval Procedures

According to the ISO 80369-6 “Small-bore connectors for liquids and gases in healthcare applications—Part 6: Connectors for neuraxial applications” standard, “neuraxial applications involve the use of medical devices intended to administer medications to neuraxial sites, wound infiltration anesthesia delivery, and other regional anesthesia procedures or to monitor or remove cerebrospinal fluid for therapeutic or diagnostic purposes”. Hence, epidural catheters are standardized under neuraxial devices. Some important parts of these are the connectors which are the components of devices that join syringes, catheters, and tubing to other medical equipment. Medical devices are frequently co-packaged or packaged in tubing sets with other devices. It is possible to administer medications or other substances to the incorrect area of the body by using the incorrect tubing. These errors, which are also referred to as tubing misconnections, wrong route errors, catheter misconnections, or Luer misconnections, may result in harm or even patient death [50,51,52].

The March 2016 release of the above ISO standard contains guidelines for creating connectors for use with neuraxial devices that are recognized by the FDA. This standard is relevant to such medical devices and is recognized for its scientific and technical merit and/or because it supports existing regulatory policies. The requirements for small-bore connectors are meant to be used for connections in neuraxial applications [51,52,53]. Moreover, small-bore connectors are used in a variety of medical devices. Small-diameter connectors are components that connect medical devices such as tubing, syringes, and other accessories to deliver liquids and gases to patients. Below is a description of the medical application or gadget and status details of the relevant international design regulations (Table 2) that come under the umbrella of the ISO 80369-6 standard [54].

## 8. Conclusions and Future Directions

In summary, we have discussed various techniques that have been developed to localize the epidural spinal needle during the lumbar puncture. Due to its critical procedure, the failure rate can reach up to 28%. Hence, these techniques that have been proposed are essential to avoid any possible side effects. The main objective was to remedy the gap between the clinical side of the problem and the engineering side by examining the important methodologies in building specific sensors for such needle localization. These techniques are based on ultrasound, mechanical, or optical schemes. The mechanical sensors record the resulting force and pressure that differ for different layers of tissues. After reviewing some of the recent research in this regard, the methods were compared. The ultrasound-guided needles offer real-time data and can help in identifying the depth and the angle of the needle, but they are relatively expensive. There is also a lack of force/pressure measured data. The other epidural needles are based on pressure measurement devices that can monitor the inserted needle pressure. They can be compact systems, but the exact kind of tissues cannot be identified and there is a need for calibration. Future development could combine pressure measurement devices with ultrasound-guided needles to overcome the lack of force/pressure measured data. The optical FBG-based devices offer dynamic data of force variation on the spinal needle with a high resolution. Finally, the required standards for the market approval of such sensors have been covered. There should be careful consideration of these standards before starting the design process. In the future, some of these sensors will potentially make such procedures easier to handle for a wide spectrum of medical professionals with fewer associated risks. However, many challenges could face such improvements as the clinical trials require a significant number of patients to be enrolled in such studies. Moreover, in order to obtain a fair comparison among these methods, there is a need to standardize the evaluation process from the perspective of the total number of punctures performed and the failure rate. Various parameters could play a significant role in such an evaluation, such as the gauge of the needle, the number of layers of the virtual tissue in ex vivo studies, the thickness of each layer, the stiffness of the layers, and the applied machine learning algorithms. 

## Figures and Tables

**Table 1:** Summary of different and recently developed techniques for epidural spinal needles.

Technology	Merits	Drawbacks
Ultrasound-guided epidural needle	- Real-time and pre-procedural ultrasound guiding of the procedure is possible using ultrasound imaging of anatomical landmarks; - The depth of the EDS and the angle of the needle insertion can both be determined by ultrasound.	- Relatively expensive; - Technical complications of co-registrations of ultrasound and epidural needle for the needle to be fully automated; - Lack of measured data for force/pressure feedback
Haptic feedback-based devices	- Enclosed in portable cases; - Connect to laptops with graphic displays.	- Lack of measured data for force/pressure feedback
Epidural pressure measurement-based devices	- Monitor and record needle-inserted pressure; - Compact system.	- Different tissues are not identified through insertions; - Needs to be calibrated.
FBG-based devices	- Dynamic data of force variation on the spinal needle with a high resolution.	- Require specific design of the FBGs.
OCT-based devices	- Image the tissue in front of the needle tip; - Deep learning models can be applied to automatically process the imaging data for needle localization.	- Relatively expensive.

**Table 2:** Summary of the relevant regulations for epidural spinal needles [54].

#	Regulation #	Device Name	Device Identification	Device Class	Ref.
1	868.5120	Anesthesia conduction catheter	A pliable tubular tool called an anesthesia conduction catheter is used to continuously administer regional anesthesia while injecting patients with local anesthetics.	Class II	[55]
2	868.5140	Anesthesia conduction kit	A tool used to deliver conduction, local, or regional anesthesia to a patient is an anesthesia conduction kit. Syringes and medications may be present in the apparatus.	Class II	[56]
3	868.5150	Anesthesia conduction needle	A patient receives regional anesthesia by having local anesthetics injected into them using an anesthesia conduction syringe.	Class II	[57]
4	880.5440	Intravascular administration set	With the aid of a needle or catheter introduced into a vein, a patient’s vascular system can receive fluids from a receptacle using an intravascular administration set. The device might consist of a needle or catheter, tubing, a flow regulator, a drip chamber, an infusion line filter, an I.V. set stopcock, fluid delivery tubing, connectors between set components, a side tube with a cap to act as an injection site, and a hollow spike to pierce and attach the tubing to an I.V. bag or other infusion fluid container.	Class II	[58]
5	880.5860	Piston syringe	A piston syringe is a tool with a calibrated hollow cylinder and a movable plunger that is used in medicine. A male connection (nozzle) for attaching the female connector (hub) of a hypodermic single-lumen needle is located at one end of the barrel. The apparatus is used to infuse or remove fluid from the body.	Class II	[59]
6	882.1620	Intracranial pressure monitoring device.	An instrument used for recording and short-term monitoring of intracranial pressures and pressure trends is known as an intracranial pressure monitoring system. The transducer, monitor, and connecting components are all part of the apparatus.	Class II	[60]
7	882.4060	Ventricular cannula	The brain’s ventricles can be punctured with a ventricular cannula in order to aspirate or introduce substances. The term “ventricular needle” is commonly used to describe this object.	Class I	[61]
8	882.5550	Central nervous system fluid shunt and components	A device or set of devices called a central nervous system fluid shunt is used to divert fluid away from the brain or another area of the central nervous system and into an internal delivery location or an external container in order to lower intracranial pressure or fluid volume (e.g., due to hydrocephalus). Catheters, valved catheters, valves, connectors, and other accessory parts are included in central nervous system shunts as well as other parts that help with shunt use or patient assessment.	Class II	[62]
9	882.4100	Ventricular catheter	A ventricular catheter is a tool used to reach the brain’s cavities so that material can be injected into or removed from the brain.	Class II	[63]
10	882.5560	Cerebrospinal fluid shunt system.	To prevent spinal cord ischemia or injury during procedures that call for lowering central nervous system pressure, a cerebrospinal fluid shunt system is a prescription device used to monitor and divert fluid from the brain or other part of the central nervous system to an internal delivery site or an external receptacle. Catheters, valved catheters, valves, connectors, and pressure sensors may be part of a cerebrospinal fluid shunt system. These components are designed to make using the shunt or evaluating a patient with a shunt easier.	Class II	[64]

## Data Availability

Not applicable.
